# Prevalence of infection in amputations in patients with diabetic foot ulcer: a retrospective study

**DOI:** 10.3389/fcdhc.2026.1746688

**Published:** 2026-04-15

**Authors:** Kaitlyn Depinet, Bryce Hockman, Rodica Muraru, David A. Ajayi, Zachary Carr, Beth Altenburger, Jaimee Haan, Gregory Westin, Emma Holler, Christopher A. Harle, Mithun Sinha

**Affiliations:** 1 Comprehensive Wound Center, Indiana University Health, Indianapolis, IN, United States; 2 Department of Surgery, Indiana University School of Medicine, Indianapolis, IN, United States; 3 Department of Health Policy and Management, Richard M. Fairbanks School of Public Health, Indianapolis, IN, United States; 4 Regenstrief Institute, Indianapolis, IN, United States; 5 Center for Diabetes and Metabolic Diseases Data and Analytics Core, Indiana University School of Medicine, Indianapolis, IN, United States

**Keywords:** amputation, diabetes, diabetic foot ulcer, infection, osteomyelitis, socioeconomic disparity

## Abstract

**Objective:**

To investigate the prevalence of infection in diabetic foot ulcers (DFUs) and its association with lower limb major and minor amputations, examining demographic and socioeconomic factors influencing DFU outcomes.

**Research design and methods:**

This retrospective study analyzed data from the Indiana Network for Patient Care (INPC) from January 2019 to May 2024. A total of 27,078 patients with DFUs were included, aged 9 to 103 years. Data included demographics, clinical encounters, ICD-10, CPT codes, laboratory values, and microbiological assessments. Analyses included Pearson correlation, logistic regression, and one-sample proportion tests.

**Results:**

Patients were predominately male (64.3%) and female (35.7%) with a mean age of 63.8 years. DFU prevalence showed significant inverse correlation with median income (r=0.2108, p=0.0016); lower-income areas (median income < $64,200) had DFU rates > 5.0 per 1,000 population. Of cultured specimens, 33.79% showed infections, primarily *Staphylococcus* spp. (22.52%) and *Streptococcus* spp. (11.27%). In major and minor amputation cases with microbial data available, *Staphylococcus* spp. (22.75%), *Enterococcus* spp. (11.86%) and *Streptococcus* spp. (10.42%) genera were most common. Osteomyelitis increased amputation odds by 7.83 times (p<0.001).

**Conclusions:**

Bacterial infections, particularly *Staphylococcus*, *Enterococcus* and *Streptococcus* genera were strongly associated with amputation risk. These findings have important clinical implications: early identification of these specific bacterial pathogens could guide targeted antibiotic therapy and inform risk stratification for amputation prevention. Improved bacterial identification could enhance DFU management and reduce complications such as amputation.

## Graphical Abstract

**Figure:**
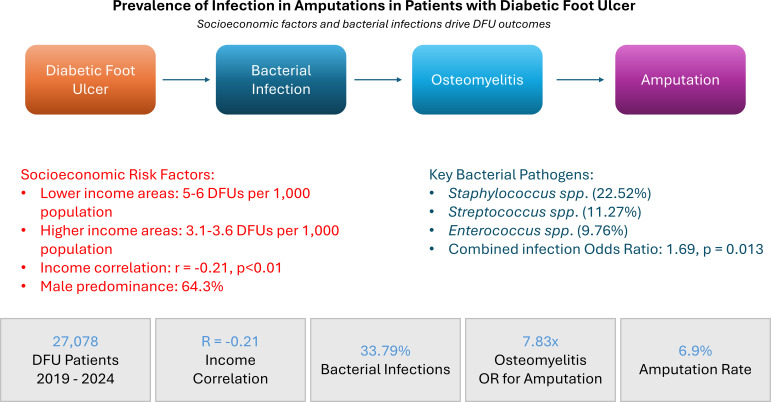

## Highlights

- Indiana DFU encounters were inversely correlated with median income, with ≥5 DFU incidence per 1,000 people in lower‑income regions.
- *Staphylococcus*, *Enterococcus*, and *Streptococcus* were the most frequent genera in amputation cases with cultures.
- Osteomyelitis increased amputation odds ~7.8‑fold after adjustment.
- Findings support risk stratification and targeted antimicrobial strategies to help prevent amputation.

## Introduction

Diabetes is a global epidemic. According to the World Health Organization (WHO), the number of adults living with diabetes worldwide has surpassed 800 million, more than quadrupling since 1990 (1). Thus around 11.1% of the world’s population live with Diabetes (2). According to the Global Burden of Diseases, injuries, and risk factors study (GBD), in 2016, about 131 million people (1.8% of the global population) had diabetes-related lower-extremity complications, including 6.8 million amputations (3). In 2022, the total cost of US healthcare towards the treatment of Diabetes was approximately $412.9 billion (4). Indiana, like many states in the US, faces significant challenges with diabetes prevalence. In a report by CDC in 2003, the state of Indiana had a age-adjusted prevalence of DFU of 16.3% among adults with diabetes which is highest in the country (5).

Diabetes has a profound and multifaceted impact on the body. When blood glucose remains elevated over prolonged periods, it can lead to a host of complications affecting various organ systems (6). Cardiovascular issues, such as heart disease and hypertension, become more prevalent (7). Diabetes also significantly impacts wound healing and increases the risk of complications, often leading to higher amputation rates (6). Amputation rates occur as a result of insufficient blood flow to the lower extremities, neuropathy reducing sensation in the foot and increasing the likelihood of developing a wound, and the risk of bacterial infection (8). Bacterial infections can set in rapidly, spread and become severe without prompt and effective treatment. Infected wounds can lead to tissue death, necessitating surgical intervention.

Our retrospective study attempts to investigate the prevalence of diabetic foot ulcer (DFU) in Indiana. From electronic medical records of Indiana Network for Patient Care (INPC) (9), one of the largest and longest running statewide health information exchanges in the US, we identified lower limb amputations, both major and minor, in patients with DFU. We also investigated the prevalence of infection in DFU and its association (if any) to lower limb amputations. Understanding the effect of infection on amputation rates is essential for improving treatment strategies and patient outcomes.

## Materials and methods

### Ethical considerations

The study protocol was reviewed and approved by the Indiana University Institutional Review Board IRB# **23582**. The IRB waived the requirement for written informed consent because all personal identifiers were removed from the dataset prior to analysis.

### Data source and study population

In this retrospective study, data were extracted from INPC (9). The INPC contains health information contributed by 80 institutions across Indiana for over 25 million patients, and its data has been used extensively in clinical and health services research. Data was provided to the research team through an honest broker service housed in the Regenstrief Institute. The INPC captures radiology reports, discharge summaries, operative notes, pathology reports, medication records, and EKG reports as their minimum contributions. INPC receives information from multiple healthcare systems. The multi-system aspect of this data is one of the big strengths as it provided us with a larger coverage of patients. We used this resource to capture DFU cases in the timeline from January 1, 2019 to May 31, 2024. A one-time data pull was conducted, with data sources specifically from healthcare institutions in Indiana, including IU Health and affiliated institutions. A total of 27,078 patients diagnosed with DFU were analyzed, ranging in age from 9 to 103 years. For the identified cases, we obtained electronic health information from the INPC and supplemental information which provided more detailed information on the cases. Global Patient Identifiers (Global Patient IDs) were utilized to ensure each patient was unique and to eliminate duplicate records during the study duration. If a patient had multiple records, we used the data associated with the earliest visit date during the study duration.

### Data collection and variables assessed

The study assessed multiple variables including: (A) demographic Information: Date of birth, height, weight, body mass index (BMI), tobacco use, race, ethnicity, and socioeconomic data based on zip code. For patients with multiple recorded values, we used the first recorded measurement within the timeline of January 1st, 2019 to May 31st 2024. Smoking status was a dichotomous variable as either Yes or No. Race and ethnicity were extracted as recorded in the electronic health registry (EHR) registration data. Patients were categorized based on sex and into the following age groups: <18, 18-39, 40-59, 60-79, and 80+ calculated at time of first DFU diagnosis. (B) Clinical Encounters: All healthcare encounters (with corresponding dates) related to DFU diagnosis, including inpatient and outpatient visits. (C) Diagnosis Data: DFU-related diagnoses and comorbidities were identified using ICD-10 codes. (D) Procedures: Amputation and any other relevant DFU-related procedures and interventions were identified through CPT codes. (E) Laboratory Values: Hemoglobin A1c (HbA1c) values with corresponding dates. (F) Medications: Prescriptions relevant to diabetes management and infection control. (G) Microbiological Assessments: Culture reports from wound specimens were collected.

### Identification of DFU, infections, and amputations

DFU cases were identified using the following ICD-10 codes (10): DFU Diagnosis (meeting either criteria I or criteria II & III): Criteria I- DFU Diagnosis codes: E10.621, E11.621, E13.621 OR criteria II- Foot ulcer diagnosis codes: L97.5X, L97.4X, L89.5X, L89.6X AND criteria III- Diabetes diagnosis codes: E10.X, E11.X, E13.X. The criteria are detailed in **Supplementary Table 1**. Bone infections were identified using the ICD-10 code M86.X. Procedure Codes for Infection Assessment: (A) Cultures with Tangential Biopsy: These were identified using CPT codes 87070 OR 87075 AND 11102. (B) Cultures without Tangential Biopsy (Swab Cultures) were identified using CPT codes 87070 OR 87075, excluding 11102. Identification of Amputations: Amputation procedures were classified as follows: (A) Major Amputations (Above the ankle): CPT codes 27882, 27884, 27886, 27880, 27889. (B) Minor Amputations (At or below the ankle): CPT codes 28810, 28820, 28825, 28112, 28880, 28126, 28805, 28122, 27888.

### Census data comparison

Demographic information like DFU distribution between age, sex, socioeconomic variables were tallied to the Indiana Census data. The Census data was based on ACS 5-Year Estimates representing aggregated (weighted average) collected from 2019 to 2023.

### Statistical analyses

For DFU demographic studies: A total of 27,078 patients diagnosed with DFU were considered. Categorical variables were summarized using frequencies and percentages. The one-sample proportion test was used to calculate the p-value for testing the age groups and sex proportions. This was used to compare these variables *vs* census population. DFU prevalence was categorized into twenty geographical areas, organized by the first three digits of ZIP codes. Utilizing the ZIP Code Tabulation Areas (2020) shapefiles from the U.S. Census Bureau, the heatmap was created. Pearson correlation was used to assess the relationship between median income per zip-code and DFU prevalence per zip code. A total of 681 zip-codes had information available for both 5-digit zip-code and median income. Other variables and cases included in the logistic regression analyses are as follows. The smoking history variable was defined as present or absent. Smoking history present included any current or past day smoker, electronic cigarette user, ex-smoker, heavy tobacco smoker, light tobacco smoker, passive smoker, smokes tobacco daily, user of smokeless tobacco. Smoking “NULL” value was considered the absence of smoking. HbA1c was used as a categorical variable. Values were classified as <8 or ≥ 8.

For microbiological studies: Bacteriology data with specified bacterial infection was available for 2194 cases. The bacteriological analyses were conducted from the first culture occurrence for each patient’s DFU. All cultures were polymicrobial. Of these 2194 cases, 319 cases also had amputations, and 1454 cases had osteomyelitis. These were used to report the most frequent species. Out of a total of 2194 cases with available bacteriology data, 21 cases were excluded due to the absence of any identified germ species. Additionally, 590 cases were excluded because of atypical BMI values (specifically a value of zero), while 46 cases were excluded for having unusual HbA1c values (either zero or exceeding 1,000). Furthermore, 19 cases were excluded due to unknown race.

For amputation studies: We had 1876 cases with amputation of which 1518 complete cases were available for the logistic regression analysis of factors associated with amputation. The Hosmer – Lemeshow goodness-of-fit test was performed to assess the logistic model. A p-value below 0.05 was considered statistically significant. STATA18 was used to perform data analyses.

## Results

### Distribution of DFU does not correlate with race but more so with the sex and socioeconomic status

A total of 27,078 patients diagnosed with DFU were included in the study. Based on their geographical distribution (zip codes), 80.32% of the patients studied reported Indiana as their residence. The minimum age was 9 years old, and the maximum age was 103 years old. The mean age was 63.80 (SD) years with a median age of 64 years. Age distribution was as follows <18 years (9; 0.03%), 18–39 years (950; 3.51%), 40–59 years (9,055; 33.43%), 60–79 years (13,755; 50.80%), and ≥ 80 years (3,309; 12.23%) (**Table 1**). One sample proportion test one-tail right side was used. The test indicated that the proportion of participants with age above or equal 40 years old was statistically significant > 50%.

**Table 1:** Age, sex, race, and ethnicity distribution in study (2019 – July 2024) vs. census data between 2019 to 2023.

Demographics	Total number in study	Study (%)	Indiana census (%)
Age			
<18	9	<1%	23.43%
18-39	950	3.51%	29.11%
40-59	9,055	33.44%	24.66%
60-79	13,755	50.80%	19.10%
80+	3,309	12.22%	3.68%
Sex			
Male	17,411	64.30%	49.56%
Female	9,664	35.69%	50.44%
Unspecified	3	<1%	--
Race			
Black/African American	3,090	11.41%	10.40%
American Indian or Alaska Native	32	1.20%	0.50%
Asian/Pacific Islander	114	4.20%	2.90%
Multiracial	817	3.02%	2.50%
White	22,269	82.25%	83.70%
Other/Unknown	756	2.78%	--
Ethnicity			
Hispanic or Latino	995	3.67%	8.8%
Not Hispanic or Latino	25,011	92.37%	76.0%
Other/Unknown	1,072	3.96%	15.2%

The cohort was predominately male (17,411; 64.30%), with 9,664 (35.69%) female and 3 (0.01%) unspecified. One sample proportion test one-tail right side was used. The test indicated that the proportion of males is statistically significant > 50%. In terms of race, most patients were White (22,269; 82.25%), followed by Black or African American (3,090; 11.41%), Multiracial (817; 3.02%), Asian/Pacific Islander (114; 0.42%), and American Indian or Alaska native (32; 0.12%), while 756 (2.78%) had race recorded as “Other/Unknown.” Ethnicity data showed that 995 (3.67%) were Hispanic or Latino, while 25,011 (92.38%) were non-Hispanic or Latino, with 1,072 (3.96%) classified as Other/Unknown (**Table 1**). We also compared race and ethnicity in the study sample with 2023 census data for Indiana. The comparison didn’t identify any racial disparity (**Table 1**).

**Figure 1:**
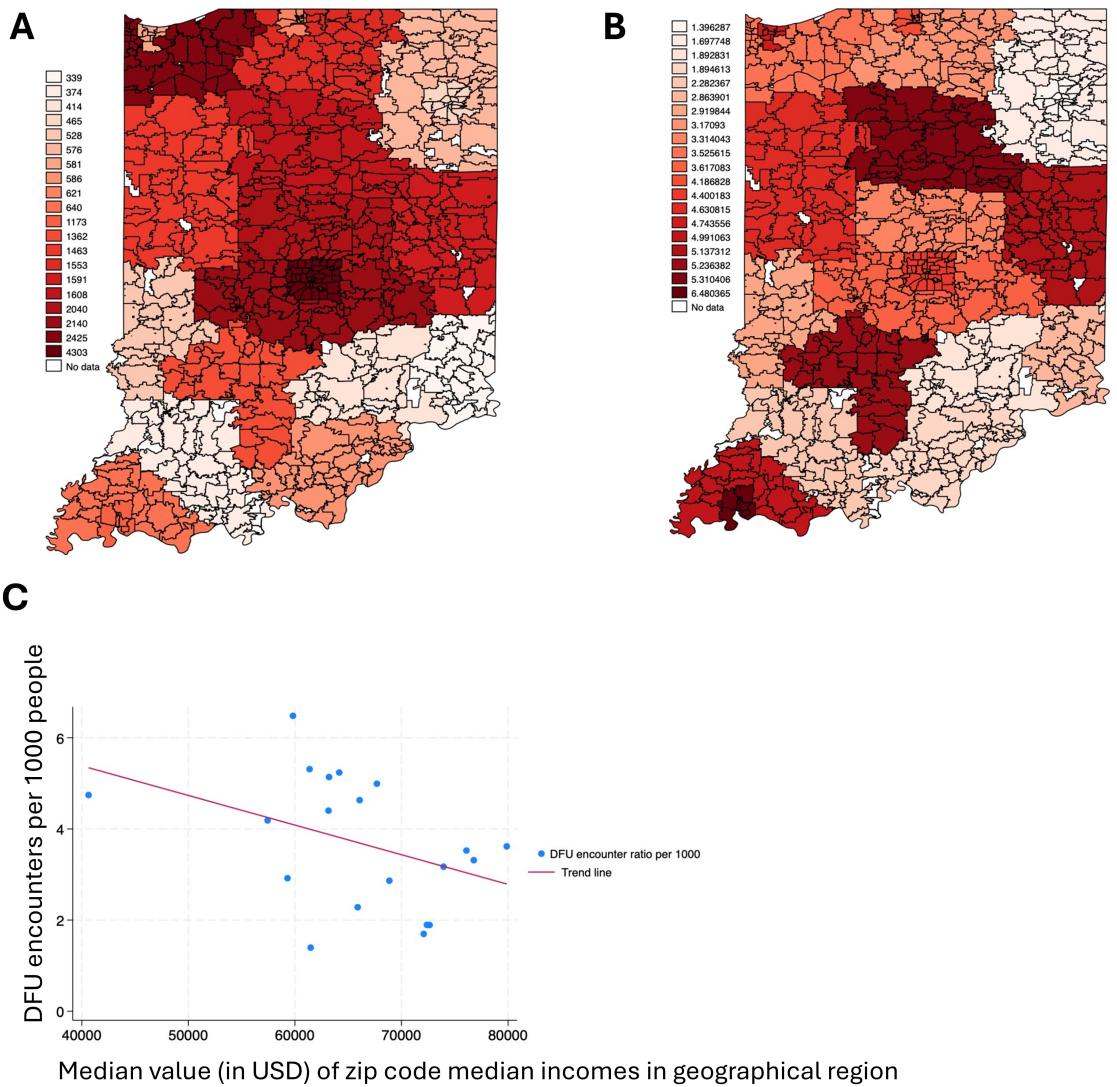
Heat map of DFU across the state of Indiana. **(A)** The total number of DFU encounters(prevalence) by geographical region, along with the defined zip code borders that compose each region. **(B)** The DFU encounters (prevalence) ratio per 1,000 people by geographical region, along with the defined zip code borders that compose each region. **(C)** A scatter plot of the DFU encounters ratio per 1,000 people in the 20 geographical areas and the median value of the individual zip codes' median incomes ($) included in the 20 geographical areas in Indiana. A downward trend line indicates a negative association.

We further inquired if the socio-economic disparity will be a better indicator of prevalence of DFU than race. To assess this, we divided the prevalence of DFU cases based on their geographical location. During this exercise, we used the first 3 digits of the zip as that is more reflective of the geographic location. Further, we divided DFU encounters with the population of the geographical location to normalize and identify the DFU prevalence estimates in that region. We preferred using the word estimate as there might be a discrepancy on data reporting to INPC by different zip codes (discussed in study limitation). We then tallied the number with the median income (as reported in census) of that geographic region. There was a statistical association between the median income and DFU encounters per region (coefficient r= -0.2108, p-value=0.0016). Results, as shown in **Figure 1**, and **Supplementary Table 2** indicate the higher prevalence of DFU in lower median income areas. For, e.g the geographical regions of 469, 473, 474 and 477 which had comparatively low median income showed prevalence of DFU of over 5 in 1000 people. The one with the lowest income of $59,000 showed over 6 in 1000 of DFU prevalence. The geographical locations with median income above $70,000 (460, 461, 463 and 465) had the lowest incidence of DFU prevalence ranging from 3.1-3.6 per 1000.

### Microbial diversity is associated with DFU

Microbial infections in DFUs are a significant concern as they can lead to poor healing outcomes. We thus investigated the abundance and diversity of microbial population in the DFU samples from the electronically archived culture reports. Among the 27,078 patients in the study, 6,669 (24.63%) had documented culture reports. Of these, 3682 (55.21%) had no mention of bacterial growth, while 2194 (32.9%) had a specified bacterial infection. An additional 201 (3.01%) had an unspecified mention of bacteria, and 592 (8.88%) showed no growth in their cultures. The prominent bacterial types identified are listed in **Tables 2**, **3**. The bacterial genera were preferred over the full species name as in some medical records species were not mentioned. It is interesting to note that *Staphylococcus* spp. (includes *S. aureus*, *S. epidermidis*), *Streptococcus* spp., *Enterococcus* spp., *Prevotella* spp., and *Bacteroides* spp. accounted for more than 50% of the cases.

**Table 2:** The prominent bacterial species in DFUs.

Bacterial species:	N (frequency)	(%)
*Staphylococcus* spp.	1,063	22.52
*Streptococcus* spp.	532	11.27
*Enterococcus* spp.	461	9.76
*Prevotella* spp.	279	5.91
*Bacteroides* spp.	257	5.44
*Proteus* spp.	243	5.15
*Pseudomonas* spp.	227	4.81
*Corynebacterium* spp.	217	4.6
*Enterobacter* spp.	169	3.58
*Peptostreptococcus* spp.	142	3.01
*Escherichia* spp.	138	2.92
*Klebsiella* spp.	133	2.82
*Morganella* spp.	69	1.46
*Citrobacter* spp.	65	1.38

Note: A total of 2194 cases with bacteriology data available entered in analysis. Patients might have multiple species.

**Table 3:** Distribution of gram-positive and gram-negative bacteria in DFUs.

Bacterial species	N (frequency)	%
Gram-positive bacteria		
*Staphylococcus* spp.	1,063	22.52
*Streptococcus* spp.	532	11.27
*Enterococcus* spp.	461	9.76
*Corynebacterium* spp.	217	4.60
*Peptostreptococcus* spp.	142	3.01
Subtotal Gram-Positive	2,415	51.16
Gram-negative bacteria		
*Prevotella* spp.	279	5.91
*Bacteroides* spp.	257	5.44
*Proteus* spp.	243	5.15
*Pseudomonas* spp.	227	4.81
*Enterobacter* spp.	169	3.58
*Escherichia* spp.	138	2.92
*Klebsiella* spp.	133	2.82
*Morganella* spp.	69	1.46
*Citrobacter* spp.	65	1.38
Subtotal Gram-Negative	1,580	33.47

Among the 10,422 patients diagnosed with osteomyelitis, 3721 (35.70%) had culture reports available. Within this subgroup, 270 (7.26%) had no mention of bacterial growth, while 1,454 (39.08%) had a specified mention of bacterial infection. Additionally, 115 (3.09%) had an unspecified mention of bacteria, and 1882 (50.58%) had no growth in their cultures. Four of the top 5 bacterial genera reported in overall DFU cases constituted in osteomyelitis as well. These included *Staphylococcus* spp., *Streptococcus* spp., *Enterococcus* spp., and *Prevotella* spp. **Supplementary Table 3**.

### Amputation in patients with DFU and its correlation with the type of DFU microbiome

Of the 27,078 patients identified with DFU, 1876 reported minor or major foot amputation during the study period. Culture results were available for 734 patients (39.13%) from the amputation set of 1876 individuals. The majority of these cultured specimens yielded either no mention of bacterial growth (343 patients; 46.73%) or identified specific bacterial pathogens (319 patients; 43.46%). Cultures with an unspecified bacterial mention were uncommon, accounting for only 19 cases (2.59%). Notably, 53 patients (7.22%) had negative culture results despite undergoing amputation. Three of the top genera reported in osteomyelitis constituted in this category of amputation with a similar ranking. These included *Staphylococcus* spp., *Enterococcus* spp., and *Streptococcus* spp. **Supplementary Table 4**. The odds of amputation was 7.83 times greater in the group with osteomyelitis compared to the reference group (those without osteomyelitis), statistically significant (p-value <0.001) after controlling for BMI, age, sex (male versus female), smoking history (positive versus non-smoker), race (African American and other race compared with White), HbA1c ≥8 (levels ≥8 compared with levels <8), and bacteriology result. Also, a positive bacteriology result for both, or three spp. among *Staphylococcus*, *Streptococcus*, and *Enterococcus* compared with other species were statistically significantly associated with amputation **Table 4**. Further, additional parameters associated with diabetes like BMI, HbA1c and neuropathy were reported for the larger set of DFU patients and subset of DFU patients who had amputations. The percentage distribution was almost similar in both the sets **Tables 5**–**7**.

**Table 4:** Risk factors associated with amputations in DFU.

Amputation	Odds ratio (OR)	p-value	95%CI
*Staphylococcus* spp./MRSA* only compared with other spp. **	1.503	0.052	[0.997-2.266]
*Streptococcus* spp. only compared with other spp. *	1.001	0.997	[0.567-1.768]
Enterococcus only* compared with other spp. *	1.473	0.161	[0.857-2.533]
both or all three among Staph/MRSA, Strep, Enterococcus* compared with other spp. **	1.693	0.013	[1.115-2.572]
Osteomyelitis present compared with absent	7.83	<0.001	[4.879-12.565]
BMI (cont.)	1	0.963	[0.983-1.017]
Age at first DFU (cont.)	1.001	0.882	[0.989-1.012]
Male compared with female	1.267	0.131	[0.932-1.724]
Smoking history compared with absence of smoking	0.906	0.478	[0.689-1.191]
Race			
African American compared with white/Caucasian	1.383	0.08	[0.962-1.987]
Another*** race compared with white/Caucasian	1.16	0.612	[0.654-2.061]
HbA1c ≥8 compared with HbA1c<8	0.933	0.632	[0.703-1.238]

Note: A total of 1518 complete cases entered in the analysis.

The Hosmer–Lemeshow test p-value = 0.51, indicating a reasonably good fit.

*Some cases might have included species from other group.

**Other spp. included the following: *Abiotrophia, Achromobacter, Acinetobacter, Actinobaculum massiliense, Actinomyces, Actinotignum, Aerococcus, Aeromonas, Alcaligenes, Anaerococcus, Arcanobacter, Arthrobacter, Atopobium, Bacillus, Bacteroides, Bifidobacterium, Butiauxella, Campylobacter, Capnocytophaga species, Citrobacter, Clostridium, Coryne, Cutibacterium, Dermabacter, Diphtheroids, Eggerthela, Eikenella, Enterobacter, Escherichia, Eubacterium, Facklamia languida, Fragilis species, Fusarium species, Fusobacter, Gemella morbillorum, Genus winkia, Globicatella, Granulicatella, Haemophilus, Hafnia, Kerstersia gyiorum, Klebsiella, Kocuria rhizophila, Lactococcus, Mobiluncus mulieris, Morganella, Neisseria, Odoribacter splanchnicus, Parvimonas, Pasteurella multocida, Peptoniphilus, Peptostreptococcus, Perfringens, Porphyromonas, Prevotella, Propionibacterium, Proteus, Providencia, Pseudomonas, Raoultella, Revotella, Rothia mucilaginosa, Serratia, Stenotrophomonas, Tissierella, Trueperella, Veillonella, Yokenella*.

***Another race included American Indian or Alaska native, Asian/Pacific islander, and multiracial

**Table 5:** Neuropathy status in study population.

Population	Neuropathy present	Neuropathy absent
Total DFU Sample (N = 27,078)	21,711 (80.2%)	5,367 (19.8%)
Amputation Cases (N = 1,867)	1,623 (86.5%)	253 (13.5%)

**Table 6:** BMI distribution in study population.

BMI category	Total sample (N = 14,368)	Amputation cases (N = 1,437)
Underweight (<18.5)	201 (1.4%)	13 (0.9%)
Healthy (18.5 – 24.9)	2,127 (14.8%)	207 (14.4%)
Overweight (25.0 – 29.9)	3,427 (23.9%)	365 (25.4%)
Obesity (≥ 30.0)	8,613 (59.9%)	852 (59.3%)

**Table 7:** HbA1c distribution in study population.

HbA1c category	Total sample (N = 22,382)	Amputation cases (N = 1,700)
HbA1c <6.5%	6,203 (27.7%)	435 (25.6%)
HbA1c 6.5 – 10.9%	13,582 (60.7%)	1,014 (59.6%)
HbA1c ≥ 11%	2,597 (11.6%)	251 (14.8%)

## Discussion

DFUs are a major source of preventable morbidity in adults with diabetes. These ulcers result in decline in functional status, infection, hospitalization, lower-extremity amputation, and even death. In consideration of the significance of the problem associated with DFU, NIH/NIDDK has established a nationwide diabetic foot consortium (11). In this retrospective study of DFU for over 5- year duration, the prevalence of DFU was found more in the males compared to females in line with published report (8). Most participants were older adults. Over 62% of DFU affected subjects were 60 years and above. Though some studies have attributed African American population to be disproportionately affected by DFU (12), we didn’t find any disproportionate overrepresentation of African-Americans in DFUs. The contribution of African American population to Indiana demographics is 10.40%, similar to 11.41% of their representation in our study. The White population stood at 83.7% similar to 82.25% of their representation in DFU.

Rather, the findings reveal a significant inverse relationship between median income and DFU prevalence. In this study, we defined the geographical region using the first three numbers of the zip code. This provided us with a broader and meaningful picture of the areas than a 5-digit zip code (600+ in Indiana) or county (100+) level data which resulted in crowding. There was a statistical association between median income and the DFU encounters per geographical region. This finding was evident in geographical locations 469, 473, 474 and 477 where median incomes were below $64,200 and DFU rates exceeded 5 per 1,000 population. Conversely, geographical regions with median incomes above $73,000 (areas 460, 461, 463, and 465) exhibited lower DFU rates of 3.1-3.6 per 1,000 population. This socioeconomic finding aligns with previous research highlighting income disparities in diabetes complications. It has been demonstrated that socioeconomic factors significantly influenced diabetes management and complication rates (8). Socioeconomic factors influence nutrition (13). Nutrition influences wound healing outcomes (14, 15). It has been reported that food deserts negatively affect wound healing outcomes (16). Systematic studies have reported the incidence of DFU in different geographical regions of the world (17, 18). Subsequent studies can be carried out to identify if geographical areas with low median income and more DFU propensity also have more food deserts. Healthcare is another factor that is directly related to socioeconomic status (19). This study could help identify if better access to wound center could be provided to the geographical areas with more DFU propensity.

DFUs are also known to frequently recur post healing (20, 21). History of bacterial planktonic and biofilm infection is often attributed to complications in wound healing and recurrence (22–24). Hence this study investigated the abundance of bacterial types available from the culture reports. The microbiological profile of DFUs in our study demonstrates consistent patterns across DFUs, osteomyelitis cases, and amputations. *Staphylococcus* and *Streptococcus* genera predominated across all categories, accounting for around 33% of all DFUs, 33% osteomyelitis cases, and 32% of amputations. Presence of two or three species among *Staphylococcus*, *Streptococcus*, and *Enterococcus* was significantly associated with increased odds of amputation compared to other species, after adjusting for demographic (age, sex, race) and clinical variables (BMI, HBA1c, smoking history). This suggests a potential role for these organisms in driving more severe disease outcomes. Both genera have been reported to be associated with DFU (25). The relevance of these bacterial genera in DFU complications both in planktonic and biofilm form has been reported through other studies as well. Additionally, Gram-negative bacteria, including Pseudomonas spp. and Escherichia spp, were identified in this study and are of substantial importance in chronic wounds due to their role in accelerating biofilm formation, which contributes to treatment resistance and poor healing outcomes (26, 27).

It is to be noted that as this is a retrospective study, we could only fetch the information that is available in the prescribed culture reports. These reports are mostly based on wound swabs as wound biopsies are not so frequently prescribed. Wound biopsy-based reports could provide information on additional bacterial types (28). Also, the aerobic cultures get more predominantly reflected due to the adopted culture conditions. This fact has been highlighted in a couple of reports on *Finegoldia magna*, an anaerobic bacterium and its pathogenic role in diabetic foot infections (29, 30). These reports highlight the lack of identification of these bacteria due to commonly employed culture techniques.

To summarize, the study suggests the variables that are important for consideration for DFU management. This study and others re-emphasize age, sex and income disparities as variables influencing DFU propensity. Focusing on the more prone geographical locations and providing them with better nutrition and healthcare could subside the incidence of DFU. A comprehensive bacterial report for aerobes and anaerobes if included in standard of care could lead to better management of DFU and prevent further complications.

### Study limitations

The period of this retrospective study was during the COVID-19 pandemic. That might have resulted in DFU encounters being on the conservative side due to reduced care-seeking during the pandemic. INPC based in Indiana is one of the largest centralized medical record databases in the country. However, regional contributions to INPC reporting differ; thus, limiting the precise frequency of DFU encounters throughout Indiana. Some regions contribute data for larger proportions of their overall population. The anaerobic bacteria were likely missed as those are not usually cultured during standard wound testing. Further, as the profiling was performed primarily with wound swabs not biopsies, some bacteria were possibly not represented in the culture report. Additionally, BMI and HbA1c variables contained numerous observations with values exceeding biologically plausible ranges, indicating potential data quality issues that warranted excluding these outliers from the regression model. This could have induced selection bias. The arterial status information was not consistently available and hence was not analyzed as a confounding variable. CPT codes not captured by INPC could have resulted in data underrepresentation. Lastly, while cases involving amputation were clearly included due to the presence of a CPT code (or any relevant medical code associated with amputation), those classified as not having an amputation were determined solely by the absence of any such medical code. This may have resulted in the inclusion of participants in no amputation category who were lost to follow-up or might have undergone amputation beyond the study duration. Due to the lack of death dates for participants who may have passed away during the study period, these individuals were not excluded from the analysis. Another limitation was the number of cases with available bacteriology data.

## Data Availability

The original contributions presented in the study are included in the article/**Supplementary Material**. Further inquiries can be directed to the corresponding author.
