# Supplementary material for: Prevalence of infection in amputations in patients with diabetic foot ulcer: a retrospective study
Source: Front Clin Diabetes Healthc. 2026 Apr 15;7:1746688. doi: 10.3389/fcdhc.2026.1746688 (PMC13125880; doi:10.3389/fcdhc.2026.1746688)
Supplement: Supplementary file 1 [file DataSheet1.docx]

**Supplementary information**

**Prevalence of Infection in Amputations in Patients with Diabetic Foot Ulcer: A Retrospective Study**

**Supplementary Table 1:**

| **Criteria** | **ICD 10 code** | **Description** |
| --- | --- | --- |
|  |  |  |
| **Criteria I** |  |  |
|  | E10.621 | Type 1 diabetes mellitus with foot ulcer |
|  | E11.621 | Type 2 diabetes mellitus with foot ulcer |
|  | E13.621 | Other specified diabetes mellitus with foot ulcer |
| **Criteria II** |  |  |
|  | L97.5x | Non-pressure chronic ulcers of other parts of the foot |
|  | L97.4x | Non-pressure chronic ulcer of heel and midfoot |
|  | L89.5 | Pressure ulcer of the ankle |
|  | L89.6 | Pressure ulcer of the heel |
| **Criteria III** |  |  |
|  | E10.X | Type 1 diabetes mellitus |
|  | E11.X | Type 2 Diabetes Mellitus |
|  | E13.X | Other specified diabetes mellitus |

**Supplementary Table 2: Diabetic Foot Ulcer (DFU) Encounter Rates by 3-Digit ZIP Code Region with Income**

| **3-Digit Geographical Location** | **Total Patients with DFU Encounters** | **Census Population** | **Median-Median Income** | **Ratio** | **Per 1,000** |
| --- | --- | --- | --- | --- | --- |
|  |  |  |  |  |  |
| 462 | 4307 | 978,823 | 63,152 | 0.0044 | 4.4 |
| 463 | 2434 | 690,376 | 76,097.50 | 0.0035 | 3.5 |
| 461 | 2140 | 591,637 | 79,877 | 0.0036 | 3.6 |
| 460 | 2045 | 617,071 | 76,786 | 0.0033 | 3.3 |
| 469 | 1609 | 302,990 | 61,377.50 | 0.0053 | 5.3 |
| 473 | 1594 | 310,279 | 63,204 | 0.0051 | 5.1 |
| 465 | 1562 | 492,600 | 73,950 | 0.0031 | 3.1 |
| 479 | 1465 | 316,359 | 66,072.50 | 0.0046 | 4.6 |
| 474 | 1370 | 261,631 | 64,162 | 0.0052 | 5.2 |
| 477 | 1188 | 183,323 | 59,821 | 0.0064 | 6.4 |

**Supplementary Table 3: Bacterial Species and Osteomyelitis Frequency**

| **Bacterial Species** | **Osteomyelitis (N)** | **%** |
| --- | --- | --- |
|  |  |  |
| *Staphylococcus spp.* | 749 | 22.39 |
| *Streptococcus spp.* | 385 | 11.51 |
| *Enterococcus spp.* | 338 | 10.1 |
| *Prevotella spp.* | 207 | 6.19 |
| *Proteus spp.* | 175 | 5.23 |
| *Bacteroides spp.* | 169 | 5.05 |
| *Corynebacterium spp.* | 167 | 4.99 |
| *Pseudomonas spp.* | 138 | 4.13 |
| *Enterobacter spp.* | 122 | 3.65 |
| *Peptostreptococcus spp.* | 96 | 2.87 |
| *Escherichia spp.* | 94 | 2.81 |
| *Klebsiella spp.* | 86 | 2.57 |
| *Morganella spp.* | 54 | 1.61 |
| *Citrobacter spp.* | 49 | 1.46 |

*Note: a total of 1454 cases with bacteriology data available entered in analysis. Patients might have multiple species*

**Supplementary Table 4: Bacterial Species and Amputation Frequency**

| **Bacterial Species** | **Amputation Culture (N)** | **%** |
| --- | --- | --- |
|  |  |  |
| *Staphylococcus spp.* | 190 | 22.75 |
| *Enterococcus spp.* | 99 | 11.86 |
| *Streptococcus spp.* | 87 | 10.42 |
| *Proteus spp.* | 55 | 6.59 |
| *Pseudomonas spp.* | 51 | 6.11 |
| *Corynebacterium spp.* | 47 | 5.63 |
| *Enterobacter spp.* | 41 | 4.91 |
| *Prevotella spp.* | 32 | 3.83 |
| *Bacteroides spp.* | 30 | 3.59 |
| *Escherichia spp.* | 23 | 2.75 |
| *Klebsiella spp.* | 21 | 2.51 |
| *Peptostreptococcus spp.* | 15 | 1.8 |
| *Citrobacter spp.* | 12 | 1.44 |
| *Morganella spp.* | 12 | 1.44 |

*Note: A total of 319 cases with bacteriology data available entered in analysis. Patients might have multiple species*
